# Tick-Borne Viruses

**DOI:** 10.1007/s12250-018-0019-0

**Published:** 2018-03-13

**Authors:** Junming Shi, Zhihong Hu, Fei Deng, Shu Shen

**Affiliations:** State Key Laboratory of Virology, Wuhan Institute of Virology, Chinese Academy of Sciences, Wuhan, 430071 China

**Keywords:** Ticks, Tick-borne viruses (TBVs), Isolation, Identification

## Abstract

Ticks are important vectors for the transmission of pathogens including viruses. The viruses carried by ticks also known as tick-borne viruses (TBVs), contain a large group of viruses with diverse genetic properties and are concluded in two orders, nine families, and at least 12 genera. Some members of the TBVs are notorious agents causing severe diseases with high mortality rates in humans and livestock, while some others may pose risks to public health that are still unclear to us. Herein, we review the current knowledge of TBVs with emphases on the history of virus isolation and identification, tick vectors, and potential pathogenicity to humans and animals, including assigned species as well as the recently discovered and unassigned species. All these will promote our understanding of the diversity of TBVs, and will facilitate the further investigation of TBVs in association with both ticks and vertebrate hosts.

## Introduction

Ticks are highly specialized obligate haematophagous ectoparasites. There are over 900 species of ticks in the world, and many of them are capable of transmitting pathogenic agents (Horak *et al.* 2002). Tick-borne viruses (TBVs) are a major risk from tick bites which could result in viral infectious diseases among animals and humans (Parola and Raoult 2001). Ticks first draw human’s intention for their infestation in animals, which would result in extensive damage to livestock health and production. The role of ticks in transmission of pathogenic viruses has been known for more than 100 years following the discovery of a flavivirus, Louping ill virus, which was identified as being responsible for severe encephalitis in sheep and other livestock (Stockman 1918). Since then, increasing numbers of TBVs have been identified, many of which are known to cause diseases in animals and humans and have been frequently reported to be associated with large epidemics. In 1957, Kyasanur Forest disease virus was first isolated during an outbreak of febrile disease in India, which caused a large number of deaths among monkeys and severe febrile illness among local residents (Holbrook 2012). Crimean–Congo hemorrhagic fever was first noted in 1945 during an epidemic among Soviet military personnel and local inhabitants (Zivcec *et al.* 2016), which was subsequently confirmed to be caused by Crimean–Congo hemorrhagic fever virus (also called Xinjiang hemorrhagic fever in China) (Butenko *et al.* 1968). Moreover, the (re-)emergence of some TBV-related epidemics or sporadic cases has also been reported in areas with a history of diseases years ago and in new geographic areas, which keeps reminding us that TBVs have always been a significant public health problem in the world.

**Fig. 1:**
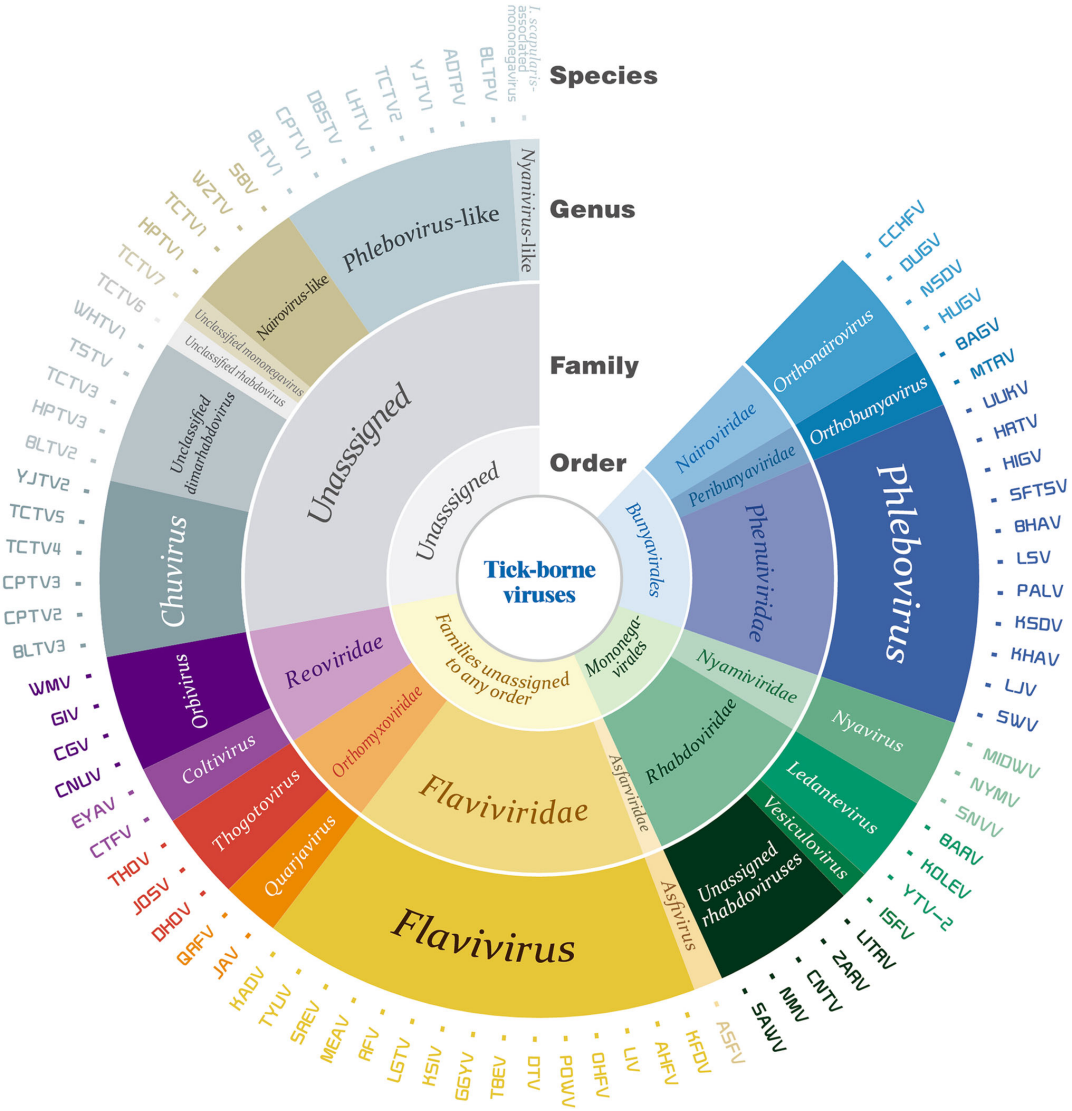
Taxonomy of tick-borne viruses. The classification of currently known tick-borne viruses was summarized in open circles which present orders, families, genera, and species of the viruses from the inner to the outer circle, respectively.

To our knowledge, the known identified TBVs include members of two orders, nine families and at least 12 genera, as well as other unassigned members (Tables 1, 2). Taking advantage of the rapid development of next generation sequencing (NGS) methods in recent years, many novel viral sequences have been identified in ticks of different species distributed in different regions of the world. Some sequences could be assigned into established families and/or genera, while many others were distantly related to known viruses and thus could not be assigned. We herein review the history of TBVs identification and isolation, classification, geographic distribution, and related diseases and epidemics, and discuss the novel viral sequences discovered in ticks recently. Viruses are described in the alphabetical order according to their taxon including *Bunyavirales*, *Mononegavirales*, and families unassigned to any order. All these suggest that ticks carry viruses of great diversities and that our current understanding of TBVs might be a tip of iceberg (Fig. 1).

**Table 1:** Classification of tick-borne viruses.

Family	Genus	Species	Main tick vectors	Geographical distributions
*Bunyavirales*				
*Nairoviridae*	*Orthonairovirus*	Crimean–Congo hemorrhagic fever virus	*Hy. marginatum*, *Ixodid* spp., *R. rossicus*	Many countries in Asia and Africa; Parts of Europe (e.g. Albania, Bulgaria)
		Dugbe virus	*Am. variegatum*, *Hy. truncatum*, *B. decoloratus*, and *R. appendiculatus*	Sub-Saharan Africa
		Nairobi sheep disease virus Ganjam virus	*R. appendiculatus*	India and Sri Lanka East and Central Africa
		Farallon virus	*Ornithodorus* spp.	USA
		Hughes virus	*O. denmarki*	USA (Florida), Trinidad, Venezuela, Cuba
		Punta Salinas virus	*O. amblus*	Peru
		Soldado virus	*O. maritimus*	North Wales, Great Britain, France, Seychelles, and Indian Ocean
		Zirqa virus	*Ornithodorus* spp., *A. cooleyi*	Abu Dhabi
*Peribunyaviridae*	*Orthobunyavirus*	*Tete orthobunyavirus serogroup*		
		Bahig virus	*Hy. marginatum*	Italy
		Matruh virus	*Hy. marginatum*	Egypt, Italy
*Phenuiviridae*	*Phlebovirus*	*Uukunimi group*		
		Uukuniemi virus	*I. ricinus*	Finland, Scandinavia, central and eastern of Europe, Azerbaijan in central Asia
		*SFTS/Heartland group*		
		Heartland virus	*Am. americanum*	USA
		Hunter island virus	*I. eudyptidis*	Australia
		Severe fever with thrombocytopenia syndrome virus	*H. longicornis*	China, South Korea and Japan
		*Bhanja group*		
		Bhanja virus	*H. intermedia*	Africa, Asia, southern Europe
		Lone Star virus	*Am. americanum*	USA (Kentucky)
		Palma virus	*H. punctate*	Portugal
		*Kaisodi group*		
		Kaisodi virus	*H. spinigera*	South India
		Khasan virus	*H. longgicornis*	Russia
		Lanjan virus	*D. auratus*	Malaya
		Silverwater virus	*H. leporispalustris*	Canada (Alberta) and USA (Wisconsin)
*Mononegavirales*				
*Nyamiviridae*	*Nyavirus*	Midway virus	*Ornithodoros* spp.	Central Pacific, Japan
		Nyamanini virus	*A. walkerae*, *A. arborerus*	Nigeria, Egypt, India, Thailand, South Africa
		Sierra Nevada virus	*O. coriaceus*	USA
*Rhabdoviridae*	*Ledantevirus*	Barur virus	*Hy. intermedia*	India, Kenya, Somalia
		Kolente virus	*Am. variegatum*	Guinea
		Yongjia tick virus 2	*H. hystricis*	China
	*Vesiculovirus*	Isfahan virus	*Hy. asiaticum*	Turkmenistan, parts of Asia
	Unassigned rhabdoviruses	Long Island tick rhabdovirus	*Am. americanum*	USA
		Zahedan rhabdovirus	*Hy. anatolicum*	Iran
		*Sawgrass virus group*		
		Connecticut virus	*I. dentatus*	USA (Connecticut)
		New Minto virus	*H. leporispalustris*	USA (East central Alaska)
		Sawgrass virus	*D. variabilis*, *H. leporispalustris*	USA (Florida)
Families unassigned to any order				
*Asfarviridae*	*Asfivirus*	African swine fever virus	*O. moubata*, *O. erraticus*	Sub-Saharan Africa, Southern Europe, South America
*Flaviviridae*	*Flavivirus*	*Mammalian tick*-*borne flavivirus group*		
		Kyasanur Forest disease virus	*H. spinigera*	India
		Alkhumra hemorrhagic fever virus	*O. savignyi*	Saudi Arabia
		Louping ill virus	*I. ricinus*	Ireland, England, Scotland, Wales
		Omsk hemorrhagic fever	*D. reticulatus*	Russia, Western Siberia
		Powassan virus	*Ixodes* spp., *I. cookei*	Canada, USA, Russia
		Deer tick virus	*I. scapularis*	New England
		Tick-borne encephalitis virus	*I. ricinus*; *I. persulcatus*, *I. ovatus*	Northern Europe Northern Asia, Siberia
		Gadgets Gully virus	*I. uriae*	Macquarie Island
		Karshi virus	*O. papillipes*	Uzbek S.S.R, North of Central Asia
		Langat virus	*I. granulatus*	Malaya
		Royal Farm virus	*A. hermanni*	Afghanistan
		*Seabird tick*-*borne flavivirus group*		
		Meaban virus	*O. maritimus*	France
		Saumarez Reef virus	*O. capensis*, *I. eudyptidis*	Australia
		Tyuleniy virus	*I. putus*	Tuleniy Island
		*Putative third group*		
		Kadam virus	*R. pravus*	Uganda
*Orthomyxoviridae*	*Quarjavirus*	Johnston Atoll virus	*O. capensis*	Australia, New Zealand and Hawaii, central Pacific
		Quaranfil virus	*A. arboreus*	Egypt, South Africa, Nigeria, Afghanistan, Kuwait, Iraq, Yemen and Iran
	*Thogotovirus*	Dhori virus	*Hyalomma* spp.	India, eastern Russia, Egypt
		Jos virus	*Amblyomma* spp. and *Rhipicephalus* spp.	Nigeria Ethiopic, Guinea, Central Africa Republic, Nigeria, Ivory Coast and Senegal
		Thogoto virus	*Rhipicephalus* spp., *Boophilus* spp., *Hyalomma* spp., and *Am. variegatum*	Central and East Africa Southern Europe, Southern Portugal
*Reoviridae*	*Coltivirus* (*Spinareovirinae*)	Colorado tick fever virus	*D. andersoni*, *D. occidentalis*, *D. albipictus*, *D. arumapertus*, *H. leporispalustris*, *Ot. lagophilus*, *I. sculptus*, and *I. spinipalpis*	USA
		Eyach virus	*I. ricinus*, *I. ventalloi*	Germany, France
	*Orbivirus* (*Sedoreovirinae*)	*Chenuda virus species*		
		Baku virus	*O. maritimus*	Caspian Sea, Uzbekistan
		Chenuda virus	*A. hermanni*	Egypt, Uzbekistan
		Essaouira virus	*O. maritimus*	Morocco
		Huacho virus	*O. amblus*	Peru
		Kala Iris virus	*O. maritimus*	Morocco
		Mono Lake virus	*A. cooleyi*	USA (Califonia)
		Sixgun city virus	*A. cooleyi*	USA
		*Chobar Gorge virus species*		
		Chobar Gorge virus	*Ornithodoros* spp.	Nepal
		*Great Island virus species*		
		Great Island virus	*I. uriae*	Canada (Newfoundland)
		Kemerovo virus	*I. persulcatus*, *I. ricinus*	Russia, Slovakia
		Lipovnik virus	*I. ricinus*	Slovakia, Czech Republic
		Tribec virus	*I. ricinus*, *H. punctata*	Slovakia, Italy, Belorussia
		St Croix River virus	*I. scapularis*, *R. appendiculatus*	N/A^a^
		*Wad Medani virus species*		
		Seletar virus	*B. microplus*	Malaysia, Singapore
		Wad Medani virus	*R. sanguineus*, *Hyalomma* spp.	East Africa, Asia, Jamaica

^a^St Croix River virus was identified from established tick cell lines. The geographic distribution of this virus was unclear to us.

**Table 2:** The unassigned viruses detected in ticks by NGS.

Viruses	Putative classification^a^	Putative tick host	Closest relative (aa identity)
Bole tick virus 3	*Chuvirus*	*Hy. asiaticum*	Midway virus (17.1%)
Changping tick virus 2	*Chuvirus*	*Dermacentor* spp.	Midway virus (17.6%)
Changping tick virus 3	*Chuvirus*	*Dermacentor* spp.	Midway virus (16.5%)
Tacheng tick virus 4	*Chuvirus*	*A. miniatus*	Midway virus (17.5%)
Tacheng tick virus 5	*Chuvirus*	*D. marginatus*	Midway virus (16.8%)
Yongjia tick virus 2	*Chuvirus*	*H. hystricis*	Nishimuro virus (54.2%)
Bole tick virus 2	*Unclassified dimarhabdovirus*	*Hy. asiaticum*	Isfahan virus (38.1%)
Huangpi tick virus 3	*Unclassified dimarhabdovirus*	*H. doenitzi*	Eel virus European X (40%)
Tacheng tick virus 3	*Unclassified dimarhabdovirus*	*D. marginatus*	Eel virus European X (39.8%)
Taishun tick virus	*Unclassified dimarhabdovirus*	*H. hystricis*	Vesicular stomatitis Indiana virus (36.6%)
Wuhan tick virus 1	*Unclassified dimarhabdovirus*	*R. microplus*	Eel virus European X (38.3%)
Tacheng tick virus 6	*Unclassified mononegavirus*	*A. miniatus*	Maize mosaic virus (20.6%)
Tacheng tick virus 7	*Unclassified rhabdovirus*	*A. miniatus*	Orchid fleck virus (24.5%)
Huangpi tick virus 1	*Nairovirus* like	*H. doenitzi*	Hazara virus (39.5%)
Tacheng tick virus 1	*Nairovirus* like	*D. marginatus*	Hazara virus (39.5%)
Wenzhou tick virus	*Nairovirus* like	*H. hystricis*	Crimean–Congo hemorrhagic fever virus (39.1%)
South Bay virus	*Nairovirus*	*I. scapularis*	Crimean–Congo hemorrhagic fever virus (37.1%)
Bole tick virus 1	*Phlebovirus*	*Hy. asiaticum*	Uukuniemi virus (37.9%)
Changping tick virus 1	*Phlebovirus*	*Dermacentor* spp.	Uukuniemi virus (37.9%)
Dabieshan tick virus	*Phlebovirus*	*H. longicornis*	Uukuniemi virus (39.2%)
Lihan tick virus	*Phlebovirus*	*R. microplus*	Uukuniemi virus (38.6%)
Tacheng tick virus 2	*Phlebovirus*	*D. marginatus*	Uukuniemi virus (39.0%)
Yongjia tick virus 1	*Phlebovirus*	*H. hystricis*	Uukuniemi virus (40.5%)
American dog tick phlebovirus	*Phlebovirus*	*D. variabilis*-associated	Precarious point virus (30.8%)
Blacklegged tick phlebovirus	*Phlebovirus*	*I. scapularis*	Precarious point virus (30.6%)
*I. scapularis*-associated mononegavirus	*Nyamiviridae*	*I. scapularis*	Midway viruses (17%)

^a^Chuvirus represents the novel group of RNA viruses which are phylogenetically related but are unassigned currently according to the results from Zhang’s study (Lin *et al.* 2015).

## *Bunyavirales*

*Bunyavirales* is a recently proposed order according to the 10^th^ ICTV report, which includes nine families. Morphological and genomic similarities like spherical virion with lipid bilayer envelope and trisegmented negative single-stranded RNA are shared by most bunyaviruses. Typical bunyaviruses are mainly arthropod-borne viruses, most of which are reported to be associated with viral diseases in vertebrates. TBVs are mainly involved in three families, *Nairoviridae*, *Peribunyaviridae* and *Phenuiviridae*.

### *Nairoviridae*

The family *Nairoviridae* has one genus *Orthonairovirus* consisting of at least 35 viruses assigned to seven serogroups as approved species (Crimean–Congo *hemorrhagic fever virus*, *Dera Ghazi Khan virus*, *Hughes virus*, *Nairobi sheep disease*, *Qalyub virus*, *Sakhalin virus*, and *Thiafora virus*) (Lasecka and Baron 2014; Walker *et al.* 2016). These nairoviruses are transmitted primarily by ticks and associated with natural hosts like birds, bats, rodents, and other animals.

The most notorious and focused upon nairovirus is Crimean–Congo hemorrhagic fever virus (CCHFV), which causes an acute febrile illness accompanied with severe bleeding named Crimean–Congo hemorrhagic fever (CCHF) in humans. CCHF was first brought to modern medical attention in 1945 when over 200 Soviet military troops and local inhabitants contracted the disease and about 10% of the patients died (Zivcec *et al.* 2016). For more than 20 years after the recognition of CCHF, researches in laboratories were limited due to the failure to isolate CCHFV. In 1968, Butenko and his colleagues isolated CCHFV via inoculating of serum from a patient in Russia into newborn mice (Butenko *et al.* 1968). CCHFV was recognized as a highly pathogenic virus to humans with a mortality rate of 30% (Guo *et al.* 2012). The recorded case of CCHFV infection presented with a wide geographic distribution including areas in western and central Asia including Xinjianng Province, China, the Middle East, south-eastern Europe and Africa (Burney *et al.* 1980; Drosten *et al.* 2002; Dunster *et al.* 2002; Fang *et al.* 2015; Nabeth *et al.* 2004). CCHFV could be transmitted by ticks in an enzootic cycle involving vertebrates and humans. Ixodid tick species from the genus *Hyalomma* and another species *Rhipicephalus rossicus* are thought to be the principle vectors for CCHFV transmission (Charrel *et al.* 2004; Hoogstraal 1979). CCHFV transmission to humans can occur via tick bite or exposure to body fluids from viremic animals or humans (Bente *et al.* 2013). Humans infected with CCHFV present non-specific symptoms, including a rapid onset high-grade fever, fatigue, and myalgia, frequently accompanied by vomiting and diarrhea. Subsequently, most patients will develop severe disease characterized by thrombocytopenia, elevated circulating liver enzymes, and hemorrhagic manifestation (Ergonul 2006; Whitehouse 2004). A fatal outcome is typically the result of disseminated intravascular coagulopathy, shock and/or multi-organ failure (Ergonul 2006; Vorou *et al.* 2007). At present, antiviral strategies to treat CCHFV infection still remain controversial or in the experimental stage. The most widely used antiviral medicine, Ribavirin, has been shown to be effective against CCHFV *in vitro* and in animal models, but its clinical benefit remains unproven (Duygu *et al.* 2012; Koksal *et al.* 2010). Dugbe virus (DUGV) belongs to CCHFV group and was first isolated from *Amblyomma variegatum* in Nigeria in 1964 (Karabatsos 1978). Then the virus was isolated from other tick species including *Hyalomma truncatum*, *Boophilus decoloratus*, *and Rhipicephalus appendiculatus*. DUGV is one of the most commonly found TBVs in Africa and is considered to be endemic in arid regions, because it was frequently isolated from ticks infesting market livestock (Sang *et al.* 2006; Burt *et al.* 1996). It can persist and replicate trans-stadially in orally infected *Am. variegatum* ticks and can be subsequently transmitted by tick feeding to a vertebrate host. As it is less pathogenic than CCHFV, but at the same time it is antigenically and genetically related to CCHFV which requires a high biosafety level laboratory, DUGV is considered an appropriate model for experimental studies on nairovirus, especially on the CCHFV infection mechanism (Coates and Sweet 1990).

Nairobi sheep disease (NSD) was first identified near Nairobi, Kenya in 1910, and Nairobi sheep disease virus (NSDV) was not found to be the causative agent until 1917 (Montgomery 1917). This virus can be transmitted by *R. appendiculatus* ticks, and causes acute hemorrhagic gastroenteritis in sheep and goats with mortality rates reaching over 90% (Idede 2000). Therefore, NSDV may cause serious damage to the farming industry. NSDV was originally thought to be endemic only in East Africa, but a recent study showed that an Asian variant of NSDV called Ganjam virus (GV), can also be found in other places including India and Sri Lanka (Dandawate *et al.* 1969; Marczinke and Nichol 2002). So to date, NSDV has been reported in a wide region including East and Central Africa, Ethiopia, Somalia, Botswana, and Mozambique. Sheep and goats are the only known vertebrate reservoirs and amplifying hosts of NSDV/GV. Human infections have also been considered because serological surveys in India were suggestive of a widespread occurrence of infection with GV (Dandawate *et al.* 1969), and human infection through a needle-stick injury, when researchers handled the virus in the laboratory, was described, which led to mild febrile illness (Rao *et al.* 1981). A study demonstrated that NSDV/GV was able to block the innate immune system (Holzer *et al.* 2011). However, compared with CCHFV, research on NSDV/GV is still limited.

The approved species *Hughes virus* includes five viruses, Farallon virus (FARV), Hughes virus (HUGV), Punta Salinas virus (PSV), Soldado virus (SOLV), and Zirqa virus (ZIRV). They are all TBVs vectored by soft ticks (Kohls *et al.* 1965). FARV was isolated from *Ornithodorus* spp. in 1965 from California, USA (Rodovsky and Stiller 1967). HUGV was first isolated from *O. denmarki* ticks collected in 1962 in Florida and subsequently from the same tick species from Soldado Rock (Gould *et al.* 1983). PSV was isolated from *O. amblus* ticks in 1967 from Peru (Converse *et al.* 1981). SOLV was isolated from *O. maritimus* in the 1960s–1970s in North Wales, Great Britain, France, Seychelles, and Indian Ocean (Chastel *et al.* 1979, 1988, 1990; Johnson *et al.* 1979). ZIRV was isolated from bird-infesting soft ticks (*Ornithodorus* spp. or *Argas. cooleyi*) in 1969 from Abu Dhabi (United Arab Emirates) (Varma *et al.* 1973). So this reveals a remarkably extensive distribution of HUGV members. However, knowledge of HUGV is limited.

### *Peribunyaviridae*

Family *Peribunyaviridae* consists of the genera *Herbevirus and Orthobunyavirus*. Genus *Orthobunyavirus* contains more than 220 viruses assigned to 48 distinct species based on serological relatedness by complement fixation test or hemagglutination inhibition and neutralization tests (https://talk.ictvonline.org/ictv-reports/ictv_online_report/). Most members of genus *Orthobunyavirus* are arboviruses transmitted by mosquitoes or culicoid flies except for a few that are vectored by ticks (Elliott 2014). At present, Bahig virus (BAGV) and Matruh virus (MTRV) within the *Tete orthobunyavirus* serogroup are the only known orthobunyaviruses transmitted by ticks (Shchetinin *et al.* 2015). The prototype of BAGV strain EgB-90 was originally isolated from the blood of *Oriolus orilus* caught at Bahig village in Egypt in 1966 (Watson *et al.* 1972). BAGV was subsequently found in migrating birds in Italy and the larvae of *Hy. marginatum* ticks on a northward migrating *Oenanthe oenanthe* in Egypt (Balducci *et al.* 1973). MTRV was first isolated from migrating passerines in Egypt, in 1961 and subsequently found in Italy from migrating birds (Balducci *et al.* 1973). Both BAGV and MTRV are vectored by *Hy. marginatum* ticks. And no human and animal disease have been associated with BAGV and MTRV yet (Hubalek and Rudolf 2012).

### *Phenuiviridae*

The family *Phenuiviridae* contains four genera: *Goukovirus*, *Phasivirus*, *Phlebovirus*, and *Tenuivirus*. Most viruses from the four genera are arboviruses. Only genus *Phlebovirus* including a large group of virus members are associated with ticks, which were usually named tick-borne phleboviruses (TBPVs).

#### *Phlebovirus*

Phleboviruses are a large group of arboviruses with transmission vectors including ticks, mosquitoes, midges, and flies. A recent study has divided phleboviruses into five phylogenetically related groups: the Sandfly/Mosquito-borne group, the Uukuniemi group, the SFTS/Heartland group, the Bhanja group, and the Kaisodi group (Matsuno *et al.* 2015). With the exception being the vectors of the Sandfly/Mosquito-borne group viruses are mainly sandflies and mosquitoes, the other groups are mostly TBPVs. The Uukuniemi group includes at least 17 species of TBPVs, which were identified in Europe, Africa, Middle Asia, Australia, and America decades ago (Palacios *et al.* 2013). The prototype of the Uukuniemi group, Uukuniemi virus (UUKV), was first isolated in 1960 from a pool of *Ixodes ricinus* ticks collected in southern Finland (Saikku and Brummer-Korvenkontio 1973). Subsequently, the virus was isolated from ticks in Scandinavia, Central and Eastern Europe, and Azerbaijan in central Asia (CDC Arbovirus catalog [http://wwwn.cdc.gov/arbocat/]). The UUKV group was not suggested to pose threat to public health, although antibodies to some members were detected in humans (Palacios *et al.* 2013).

In recent years, newly emerging TBPVs able to induce severe diseases in humans have attracted increasing attention. Severe fever with thrombocytopenia syndrome virus (SFTSV) is one of the novel pathogenic TBPVs and was first identified in China from patients with severe fever, thrombocytopenia, and leukocytopenia accompanied with gastrointestinal symptoms, chills, joint pain, and myalgia (Fang *et al.* 2015; Yu *et al.* 2011). The disease is so called severe fever with thrombocytopenia syndrome (SFTS) with an initially reported fatality rate of up to 30% (Yu *et al.* 2011). Some patients reported a history of tick bite, and SFTSV was detected mainly in *Haemaphysalis longicornis* ticks from where the patients lived (Luo *et al.* 2015). Human-to-human transmission through direct blood contact was also reported in clusters of SFTS patients (Bao *et al.* 2011; Liu *et al.* 2012), and other potential transmission routes among persons were also supposed but need further investigation (Gong *et al.* 2015; Huang *et al.* 2017; Jeong *et al.* 2016). From 2010 to 2016, 23 provinces in China have reported over 10,000 SFTS cases with a mean mortality rate of 5.3% (Zhan *et al.* 2017). After SFTSV identification in China, SFTSV infection was also reported in Japan and South Korea (Hiraki *et al.* 2014; Kim *et al.* 2013; Yoo *et al.* 2016; Yoshikawa *et al.* 2015). Phylogenetic analyses showed that the genotypes of SFTSV strains were associated with geographic distributions (Shi *et al.* 2017; Yoshikawa *et al.* 2015). Recombination and reassortment had happened among different strains and genotypes due to the migration of SFTSV from different locations, which might result in increasing genetic diversity of SFTSV (Liu *et al.* 2014; Shi *et al.* 2017). Heartland virus (HRTV) was first isolated from two patients in Missouri, USA, who had diseases with similar symptoms to SFTS (McMullan *et al.* 2012). HRTV shares similarity of about 60%–70% to SFTSV, and was phylogenetically related to SFTSV (McMullan *et al.* 2012). Detection of HRTV RNA and isolation of HRTV from *Am. americanum* ticks revealed *Am. americanum* may be the primary vector for HRTV transmission. To date, over 30 cases of HRTV infection, including two deaths, have been reported in the United States (Bosco-Lauth *et al.* 2015; Fill *et al.* 2017; Muehlenbachs *et al.* 2014; Riemersma and Komar 2015; Westover *et al.* 2017), while no other countries nor territories have reported cases associated with HRTV (Savage *et al.* 2013). So far, studies on the pathogenesis of both SFTSV and HRTV are limited, as virus infection could not induce significant clinical signs in experimental animals. Several autopsy cases with SFTSV and HRTV infection were reported from Japan and America, reflecting different pathological findings but this was probably due to the personal variations of the deaths (Fill *et al.* 2017; Hiraki *et al.* 2014; Muehlenbachs *et al.* 2014). Hunter Island group virus (HIGV) was isolated from *I. eudyptidis* ticks collected from a village in Australia (Wang *et al.* 2014). However, HIGV was not suggested to be related to the disease in the village where an outbreak occurred in 2002. Phylogenetic analyses showed that HIGV, HRTV, and SFTSV constitute the SFTS/Heartland group, indicating that HIGV might be a pathogen with zoonotic potential (Matsuno *et al.* 2015; Wang *et al.* 2014).

Since the identification of TBPVs related to human diseases, increasing attention has been paid to other TBPVs that were identified years ago but have not been characterized yet. Bhanja virus (BHAV) was first isolated from *H. intermedia* ticks in India in 1954 and then in Africa and Europe (Hubalek 1987b; Hubalek *et al.* 1988). It was confirmed as a neurotropic virus to be able to cause disease in ruminants and humans (Balducci *et al.* 1970; Hubalek 1987a). Palma virus (PALV) was isolated from *H. punctata* ticks in Portugal in 1992 (Filipe *et al.* 1994). The phylogenetic relationships of BHAV and PALV were not clarified until 2012, when the complete genome of both viruses was sequenced. The results suggested that BHAV and PALV are novel TBPVs distinct from Uukuniemi group and the SFTS/Heartland group, and form a new group termed as the Bhanja group (Matsuno *et al.* 2015). Lone star virus (LSV) was originally isolated from *Am. americanum* (the lone star tick) in Kentucky in 1967 (Kokernot *et al.* 1969), and was unclassified until 2013 when the sequence was reported (Swei *et al.* 2013). LSV can infect human (HeLa) and monkey (Vero) cells, but no evidence for human infection has been reported (Labuda and Nuttall 2004). Phylogenetic analysis showed that LSV also belonged to Bhanja group (Matsuno *et al.* 2015).

The Kaisodi group includes four members, including Kaisodi virus (KSDV) from *H. spinigera* ticks in 1957 (Bhatt *et al.* 1966), Khasan virus (KHAV) from *H. longicornis* ticks in 1971 (Al’khovskii *et al.* 2013), Lanjan virus (LJV) from *Dermacentor auratus* ticks in 1960 (Tan *et al.* 1967), and Silverwater virus (SWV) isolated from *H. leporispalustris* ticks in 1960 (Hoff *et al.* 1971). After the full-length genomes of these viruses were obtained recently, benefiting from NGS, these viruses have been identified as being novel TBPVs (Matsuno *et al.* 2015). So far, no infectious cases have been reported to be associated with members of the Kaisodi group.

## *Mononegavirales*

The viral order *Mononegavirales* accommodates viruses with nonsegmented, linear, single-stranded negative-sense RNA genomes (Afonso *et al.* 2016). Two families in *Monogegavirales*, including *Nyamiviridae* and *Rhabdoviridae*, contain members of TBVs.

### *Nyamiviridae*

The family *Nyamiviridae* is a newly proposed taxon belonging to *Mononegavirales*, which currently consists of three genera (*Nyavirus*, *Peropuvirus*, and *Socyvirus*) (Kuhn *et al.* 2013a, b). Three species in genus *Nyavirus* are TBVs, Midway nyavirus (MIDWV), Nyamanini nyavirus (NYMV), and Sierra Nevada nyavirus (SNVV) (Kuhn *et al.* 2013a). MIDWV was first isolated in 1966 from seabird ticks *Ornithodoros* spp. collected on the Midway, Kure, and Manana islands in the Central Pacific and from northern Honshu, Japan. Additionally, two species of nestling seabirds, *Larus crassirostris* and *Nycticorax nycticorax*, were found to have specific antibody to MIDWV. MIDWV was confirmed to be pathogenic to newborn Swiss mice. (Takahashi *et al.* 1982). NYMV was first isolated in 1957 from a cattle egret in South Africa (Taylor *et al.* 1966b). Then it was then repeatedly isolated from cattle egrets and *A.* *walkerae* soft ticks in Nigeria, Egypt, India, and Thailand (Kaiser 1966; Kemp *et al.* 1975; Taylor *et al.* 1966b). NYMV is considered primarily a bird virus and postulated to be transovarially transmitted by *A. arborerus* ticks (Kaiser 1966). No human infection or disease was associated with NYMV. Suckling mice succumbed to NYMV infection 7 days after intracerebral inoculation, and NYMV can productively infects a variety of mammalian cells (Kaiser 1966). MIDWV and NYMV are antigenically related to each other, but they have different geographic distribution and host spectrum (Takahashi *et al.* 1982). Both viruses are negative-stranded RNA viruses sharing ~ 63% genomic identity, and show a high genetic divergence from all other tested viruses (Mihindukulasuriya *et al.* 2009). They were therefore proposed to be two novel virus species, creating the novel genus *Nyavirus* (Kuhn *et al.* 2013b). SNVV was first isolated in 1975 from soft ticks *O. coriaceus* collected during the investigation of an outbreak of epizootic bovine abortion (EBA) in northern California, USA. However, it was not the causative agent of EBA. SNVV has about 50% similarity to NYMV and MIDWV, and is phylogenetically related to them (Rogers *et al.* 2014). It is presently unknown whether SNVV naturally infects birds or mammals.

### *Rhabdoviridae*

The family *Rhabdoviridae* is composed of a large and diverse group of viruses that can infect a wide range of vertebrates, invertebrates, and plants (Kuzmin *et al.* 2009). According to the latest official report of ICTV in 2016, the family *Rhabdoviridae* can be taxonomically classified into 18 genera (*Almendravirus*, *Curiovirus*, *Cytorhabdovirus*, *Dichorhavirus*, *Ephemerovirus*, *Hapavirus*, *Ledantevirus*, *Lyssavirus*, *Novirhabdovirus*, *Nucleorhabdovirus*, *Perhabdovirus*, *Sigmavirus*, *Sprivivirus*, *Sripuvirus*, *Tibrovirus*, *Tupavirus*, *Varicosavirus*, and *Vesiculovirus*) and other unassigned rhabdoviruses. The virions of rhabdoviruses are characteristically bullet-shaped particles with the length of 100–430 nm and the diameter of 45–100 nm (Dilcher *et al.* 2015). Rhabdoviruses are non-segmented, negative-sense RNA viruses with 11–15 kbp genomes coding for at least five transcription units: nucleoprotein (N), phosphprotein (P), matrix protein (M), glycoprotein (G) and the RNA-dependent RNA polymerase (RdRp) (King *et al.* 2012). Rhabdoviruses are mainly transmitted by arthropod vectors, and a few are TBV species which are summarized below.

#### *Ledantevirus*

Genus *Ledantevirus* comprises 14 species. Three of them are TBVs, including Barur virus (BARV), Kolente virus (KOLEV), and Yongjia tick virus 2 (YTV-2) (Kazimirova *et al.* 2017). BARV was isolates from *Hy. intermedia* ticks in India, Kenya, and Somalia (Butenko *et al.* 1981; Johnson *et al.* 1977). Monkeys could be infected by BARV, which induced typical localization of lesions in the brain and resulted in the death of the experimental monkeys (Abramova *et al.* 1987). KOLEV was firstly isolated in Guinea in 1985 from Jones’s leaf-nosed bat and a pool of ticks (*Am. variegatum*) (Butenko 1996; Konstantinov *et al.* 2006). Significant cytopathic effect (CPE) could be induced by KOLEV infection in baby hamster kidney (BHK-21) cells. Newborn mice inoculated intracranially with KOLEV showed signs of illness including loss of balance, paralysis, and lethargy (Konstantinov *et al.* 2006). YTV-2 was identified by NGS from hard ticks (*H. hystricis*) collected from wild and domestic animals in Zhejiang Province, China, however, the virus was not isolated. Phylogenetic analysis showed that YTV-2 shares high amino acid similarity to all other ledanteviruses (Li *et al.* 2015). Little is known about these tick-borne rhabdoviruses in association with human or animal disease (Ghedin *et al.* 2013).

#### *Vesiculovirus*

The members of genus *Vesiculovirus* are mainly arthropod-borne viruses that are transmitted by biting insects, most likely sandflies (Nasar *et al.* 2017). The Isfahan virus (ISFV) is the unique member in genus *Vesiculovirus* that has been isolated from *Hy. asiaticum* ticks in Turkmenistan (Kazimirova *et al.* 2017; Labuda and Nuttall 2004). It was first isolated from phlebotomine sandflies in Isfahan Province, Iran in 1975, and is endemic in parts of Asia, including Iran, Turkmenistan and the central Asia republics (Tesh *et al.* 1977). Serological surveys in Iran following the isolation of ISFV detected neutralizing antibodies in humans and in rodents but not in sheep, goats, cattle, chickens or pigeons. Such results suggest that ISFV is not readily transmitted to the latter species and is very likely restricted to a limited range of hosts (Wilks and House 1986). Infection by ISFV has not been linked to any illness in natural infection among domestic animals and human (Gaidamovich *et al.* 1978). Compared with other vesiculoviruses much milder illness was induced by ISFV in the experimental infection of domestic animals, including pony, steer, sheep, goat and pig (Wilks and House 1986).

#### *Unassigned rhabdoviruses*

The only other rhabdoviruses isolated from ticks are all unassigned to any genera presently including two genetically related viruses (Long Island tick rhabdovirus [LITRV] and Zahedan rhabdovirus [ZARV]), and the Sawgrass virus group containing three TBVs.

LITRV was identified in long star ticks (*Am. americanum*) collected in New York in a program for viral surveillance and discovered in ticks through high-throughput sequencing (HTS) (Tokarz *et al.* 2014a). ZARV was isolated from *Hy. anatolicum* ticks from Iran (Dilcher *et al.* 2015). However, sequence comparisons and phylogenetic analyses do not associate LITRV and ZARV with any one of the recognized species or genera of this family, but these two viruses form a monophyletic clade with an unassigned mosquito-borne rhabdovirus from Côte d’Ivoire, which is proposed to be a unique taxonomic group within *Rhabdoviridae* (Tokarz *et al.* 2014a). ZARV can cause typical CPE when inoculated on Vero cells, but no disease developed after subcutaneous and intraperitoneal inoculation into newborn mice. However, important questions about the role of ticks in enzootic transmission of LITRV and ZARV, and the pathogenicity of these viruses to mammalian hosts are unanswered (King *et al.* 2012; Tokarz *et al.* 2014a).

The Sawgrass virus group consists of Connecticut virus (CNTV), New Minto virus (NMV), and Sawgrass virus (SAWV), which were first isolated years ago in *D. variabilis* ticks, *H. leporispalustris* ticks, and *I. dentatus* ticks from the USA, respectively (Ritter *et al.* 1978; Sather *et al.* 1970; Walker *et al.* 2015). However, further studies are required to clarify whether ticks can transmit these viruses and their pathogenicity to humans and animals.

## Families Unassigned to Any Order

### *Asfarviridae*

The family *Asfarviridae* has one genus, the genus *Asfivirus*, and comprises only single species, African swine fever virus (ASFV). It is the only known DNA arbovirus transmitted by ticks, and could infect and cause disease in swine. The African swine fever disease (ASF) caused by ASFV was first identified in Kenya in the 1920s (Montgomery 1921). At this point, it was confined to Africa, but then spread to Europe in the middle of the last century, and later to South America and the Caribbean (Galindo and Alonso 2017). ASF was eradicated from Europe, except for Sardinia, in the 1990s via drastic control and eradication programs. However, in 2007, the disease spread again out of Africa into Caucasus, especially Georgia, and in 2014 it reached the eastern territory of the European Union (EU). The latest reports of the disease include an increasing list of EU countries, Poland and the three Baltic republics and Moldova (Pejsak *et al.* 2014; Wozniakowski *et al.* 2016). This re-emerge may be a consequence of the ASFV increase in Africa in combination with the globalization and usage of contaminated garbage/swill to feed pigs (Sanchez-Vizcaino *et al.* 2013).

ASFV isolates differ in virulence, and may produce acute, chronic, or even inapparent infections. Virulent isolates can cause 100% mortality within 7–10 days, while less virulent isolates may produce a mild disease. A number of infected swine were found to become ASFV carriers after they recovered from the disease (Vinuela 1985). Soft ticks from the genus *Ornithodoros* are the main vectors of ASFV. *O. moubata* ticks are involved in the sylvatic transmission cycle of ASFV in sub- Saharan Africa, and *O. erraticus* is involved in Europe (Anderson *et al.* 1998). ASFV can be transmitted in tick trans-stadially, transovarially, and sexually (Kleiboeker *et al.* 1999). It can also be transmitted by a direct oral route in wart hogs, giant forest hogs, and bush pigs, and results in asymptomatic disease or disease with persistent infection, while high mortality and hemorrhage in domestic and wild pigs is usually related to ASFV infection (Detray 1957). Transmission between domestic animals can also occur by ingestion of infected meat and fomites, or mechanically by biting flies (Costard *et al.* 2013; Guinat *et al.* 2016). A striking feature of ASFV infections is the absence of neutralizing antibody production. This has severely hampered attempts to produce an effective vaccine although the use of gene deleted virus strains has shown promise in protecting against virulent strains (Zakaryan and Revilla 2016).

### *Flaviviridae*

Family *Flaviviridae* consists of four genera, *Flavivirus*, *Hepacivirus*, *Pegivirus*, and *Pestivirus* (Fukuhara *et al.* 2017). The genomes of *Flaviviridae* viruses commonly contain a single-strand positive RNA encoding a polyprotein which will be cleaved into 2–4 structural proteins and 7–9 non-structural proteins (Chambers *et al.* 1990). The genus *Flavivirus* is a large group of arboviruses able to infect many vertebrates, they can be transmitted by mosquitos, ticks, or specific arthropod vectors. According to the different types of vectors, flaviviruses can be divided in to the tick-borne flavivirus (TBFV) group, the mosquito-borne flavivirus (MBFV) group, and the no known vector group (NKV) (Valarcher *et al.* 2015; Weaver and Barrett 2004). At least 12 species of TBFVs currently have been recognized and divided into the mammalian tick-borne flavivirus group (M-TBFV) and seabird tick-borne flavivirus group (S-TBFV) (Gritsun *et al.* 2003). In the M-TBFV group, six important pathogens of humans or animals are known as the “tick-borne encephalitis (TBE) serocomplex” including Kyasanur Forest disease virus, Louping ill virus, Omsk hemorrhagic fever virus, Powassan virus, and Tick-borne encephalitis virus. The Kyasanur Forest disease virus and Omsk hemorrhagic fever virus cause hemorrhagic fever in humans, while the others are encephalitic viruses (Grard *et al.* 2007).

Tick-borne encephalitis virus (TBEV) is the most notorious members of genus *Flavivirus*. It is the etiological agent of a severe human neurological infectious disease (Tick borne encephalitis, TBE). Terrible neurological symptoms were observed in patients with severe TBE, including muscle paralysis, disturbance of consciousness, and difficulties in swallowing, and verbal communication. Torturous sequelae were quite common among severe TBE patients (Solomon *et al.* 2007). Nearly half of the patients of TBE reported difficulties with memory and concentration (Gritsun *et al.* 2003; Marjelund *et al.* 2004). TBE was first discovered by Soviet scientists in the 1930s in the Far East under extremely harsh conditions. Some of the scientists paid for this pioneering work with their health and even lives (Zlobin *et al.* 2017). Since then, areas ranging from northern China and Japan, through far-eastern Russia to Europe have been afflicted by thousands of human cases of TBEV infection each year (Dumpis *et al.* 1999; Gould *et al.* 2006). TBEV can be classified into three genotypes in association with their geographic distributions, the European (TBEV-Eu), Siberian (TBEV-Sib), and Far-eastern (TBEV-FE) subtypes. The TBEV-Eu subtype is predominantly epidemic in Europe and Russia; the TBEV-Sib subtype is mainly present in Siberian in Russia; and the TBEV-FE subtype is endemic mainly in China, Japan and far-eastern Russia. Other areas like Lithuania, Sweden, and Denmark, where no TBE cases have been reported, were confirmed to have TBEV-specific antibodies circulating in animal and human populations (Paulsen *et al.* 2015; Pettersson *et al.* 2014). The transmitting vectors of TBEV are verified according to the subtypes. The TBEV-Eu subtype is mainly transmitted by *I. ricinus*, while TBEV-FE and TBEV-Sib are associated with *I. persulcatus*. Wild mammalian hosts are also involved in the maintenance and circulation of TBEV in nature, especially rodents which act as maintenance and amplifying hosts and the reservoir hosts for TBEV (Suss 2003). Human infections with the TBEV-FE are usually more severe than infection with the other two subtypes, with more frequent encephalitis signs and a higher fatality rate estimated as 5%–35%, compared with 1%–2% for TBEV-Eu and 6%–8% for TBEV-Sib (Gritsun *et al.* 2003). Presently, only the Far Eastern subtype is endemic in China (Gao *et al.* 2010; Lu *et al.* 2008). Several provinces including Xinjiang, Tibet, Inner Mongolia, Jilin, Heilongjiang, Sichuan, and Yunnan have reported cases of TBEV infection (Zhang *et al.* 2013; Zhao *et al.* 2012). *I. persulcatus* ticks are the predominant tick species in China and serve as the major transmission vector of TBEV (Lindquist and Vapalahti 2008; Sun *et al.* 2017). Other tick species like *I. ovatus* is responsible for TBEV transmission in Yunan Province (Lu *et al.* 2008).

The virus species *Kyasanur Forest disease virus* consists of two subtypes: Kyasanur Forest disease virus (KFDV) and Alkhumra hemorrhagic fever virus (AHFV). KFDV is the prototype of this species and is a zoonotic tick-borne viral disease that causes significant morbidity and mortality in human and monkey populations. It was known to exist in Kyasanur Forest of Karnataka State, India, since 1957 and has spread beyond into surrounding states (Awate *et al.* 2016; Work *et al.* 1957). Variants have also emerged in Saudi Arabia and Egypt and possibly in China (Musso *et al.* 2015; Wang *et al.* 2009). KFDV is maintained in nature through small mammals, shrews, bats, and monkeys and also in ticks (Pattnaik 2006). It is mainly transmitted to monkeys and humans through tick bites (Sreenivasan *et al.* 1986). The number of monkeys affected by this disease has increased substantially in the past few decades (Awate *et al.* 2016; Jeffries *et al.* 2014). In India, human cases with KFDV infection have been continuously reported. Especially in 2014 and 2015, human/monkey deaths associated with KFDV infection were identified (Sadanandane *et al.* 2017). Annual numbers of human cases of KFDV are estimated at 400–500 with a fatality rate of 3%–5% (Holbrook 2012). The status of KFDV as a high-risk containment level-4 agent has restricted research on its pathogenicity and the development of therapeutics (Cook *et al.* 2016). AHFV was first isolated from the blood of six male butchers in Saudi Arabia in 1995, among who two died and the other four recovered. All AHFV infected patients had similar manifestations including fever, headache, generalized body aches, arthralgias, anorexia, vomiting, leucopenia, thrombocytopenia, elevated liver enzymes, elevated creatinine phosphokinase, and elevated blood urea. It was suggested that the human infections by AHFV may be related to contact with infected sheep or ticks feeding on sheep (Dodd *et al.* 2011; Zaki 1997). Genetic analysis showed that AHFV was highly related to KFDV and it was recommended as a subtype of KFDV (Charrel *et al.* 2001).

Louping ill disease is a tick borne viral infection that predominantly affects sheep causing neurological disease with the reported morbidity ranging from 5% to 60% in different areas. Typical manifestations included diffuse non-suppurative meningoencephalitis with ataxia, pyrexia, seizures and opisthotonus, posterior paralysis, coma, and death (Scott *et al.* 2007). Louping ill virus (LIV) is the causative agent which was first isolated in Scotland in 1929 and was the first isolated arthropod-borne virus in Europe (Doherty *et al.* 1971). It is mainly detected in sheep, cattle, red grouse and ticks in upland areas of the British Isles, particularly in Scotland, Cumbria, Wales, Devon and Ireland (Jeffries *et al.* 2014). LIV was also detected in other animals, including goats, dogs, pigs, horses, deer, llamas, alpacas, and mountain hares (Jeffries *et al.* 2014; Reid *et al.* 1978; Timoney *et al.* 1976). *I. ricinus* is the major transmission vector and is associated with the distribution of LIV (Gilbert 2010). Seasonal occurrence, presenting with a high prevalence in spring and autumn, is consistent with the activity of the tick vector (Randolph *et al.* 2002). The first incidence of possible human LIV infection was reported in 1934, and since then there have been 44 further published reports of clinical disease in man. Most reported LIV infections occurred through occupational exposure to infected livestock including in stockmen, abattoir workers, butchers, and veterinarians who have frequent contact with sheep or other potentially infectious species (Williams and Thorburn 1962).

Omsk hemorrhagic fever virus (OHFV) was identified to be the causative agent of Omsk hemorrhagic fever (OHF) which was first diagnosed in 1940s in Russia. OHFV was first isolated from a patient’s blood in 1947, and later from *D. reticulatus* ticks, muskrats, and other vertebrates and arthropods. OHFV is genetically related to TBEV, but was suggested to be a unique species based on antigenic tests (Gaidamovich *et al.* 1989). In contrast to TBEV infection, OHFV infection does not invade the central nervous system, but results in mild flu-like symptoms. OHFV infection also causes hemorrhage, including nosebleeds, bleeding gums, vomiting of blood, blood in the lungs, and non-menstrual bleeding of the uterus. The case-fatality rate varies from 0.5% to 2.5%, and recovery from OHF is generally slow (Ruzek *et al.* 2010). Different to the widespread of TBEV, OHFV had remained restricted to four Siberian provinces during hundreds of years of evolution (Karan *et al.* 2014). It is still unclear why the OHFV endemic area is much smaller than where the major vectors and hosts (*Dermacentor* spp. ticks and muskrats) are distributed.

Powassan virus (POWV) is a tick-borne flavivirus transmitted by *Ixodes* spp. and can cause a fatal neuroinvasive disease in human. The virus was considered as a human pathogen when it was first isolated from the brain of a young boy who died of encephalitis in the town of Powassan (Canada) (McLean and Larke 1963). Since then, human cases of POWV have been documented in the United States, Canada, and Russia. The majority of symptomatic POWV cases typically involve an initial febrile illness. During the primary phase, sore throat, drowsiness, headache, and disorientation are commonly present (Smith *et al.* 1974). In severe cases, the most common clinical presentations of disease are encephalitis, meningoencephalitis, and aseptic meningitis (Smith *et al.* 1974). Approximately 10% of POWV encephalitis cases are fatal, and severe and long-lasting neurological sequelae are present in over 50% of survivors (Ebel 2010). In recent years, the incidence of human infection of POWV appears to be rising probably due to the enhanced surveillance and testing for arthropod-borne viruses or actual emergence of the disease (Ebel *et al.* 2001; Hinten *et al.* 2008; Piantadosi *et al.* 2016). However, compared with other human pathogenic flaviviruses, POWV has been overlooked. POWV was considered as a TBE serocomplex because of the high similarity to TBEV. Much of the understanding of POWV comes from research conducted with TBEV (Kuno *et al.* 2001). In 1997, a novel virus that was similar but distinct from POWV was isolated from *I. scapularis* tick (deer tick) in New England (USA) and was named deer tick virus (DTV) (Telford *et al.* 1997). Sequence alignment revealed a high level of genetic similarity between DTV and POWV, with 84% nucleotide sequence identity and 94% amino acid identity. After that, the POWV strains were divided into two genetic lineages or genotypes, the POWV lineage and the DTV lineage (Beasley *et al.* 2001). Although experimental evidence from mouse infection was suggestive that DTV was less infectious than other members of the TBE serocomplex viruses including TBEV and LIV (Telford *et al.* 1997), it could cause neuroinvasiveness in mice similar to POWV infection in humans. So the public significance of DTV deserves further investigation (Beasley *et al.* 2001).

Four other M-TBFVs, Gadgets Gully virus (GGYV), Karshi virus (KSIV), Langat virus (LGTV) and Royal Farm virus (RFV) are not considered human pathogens at present. Knowledge about their ecology and epidemiology is limited. GGYV was first isolated from pools of seabird ticks (*I. uriae*) collected between 1975 and 1979 at Macquarie Island (St George *et al.* 1985). After near 30 years, a new strain with 96% identity to the first one was isolated from *I. uriae* ticks collected in the same island, suggesting this flavivirus has maintained remarkable genetic stability for over 30 years. Phylogenetic analysis showed that GGYV was more related to the M-TBFV groups although it was discovered from seabird ticks (Major *et al.* 2009). KSIV was first isolated from *O. papillipes* ticks collected in 1971 from Karshinsk steppe, Uzbekistan (Lvov *et al.* 1976). Subsequently, it was isolated from *H. asiaticum* ticks collected in the north of Central Asia (Alma-Ata region of the Kazakh Soviet Socialist Republic) in 1976 (Khutoretskaya *et al.* 1985). KSIV was experimentally proved to be transmitted both by ticks and mosquitoes (Khutoretskaya *et al.* 1985). So far, it is not known to cause disease in humans (Grard *et al.* 2007). LGTV was firstly isolated via intracerebral inoculation into suckling mice from a pool of hard ticks (*I. granulatus*) from forest rats caught near Kuala Lumpur in Malaya. LGTV can cause paralysis in adult mice and induce fever in macaque monkeys (Smith 1956). No human disease has been reported to be associated with LGTV (Campbell and Pletnev 2000). RFV was isolated from *A. hermanni* ticks collected in Afghanistan. Complement fixation testing showed it was closely related to POWV. The virus was pathogenic for hamster but not for weanling guinea pigs and rabbits (Williams *et al.* 1972).

Seabirds are hosts of at least 29 tick species and thus may play roles in dispersal of virus due to their large population size, wide geographic distributions, and high mobility (Dietrich *et al.* 2011). The S-TBFV group includes three species: Meaban virus (MEAV), Saumarez Reef virus (SREV), and Tyuleniy virus (TYUV). MEAV was first isolated from *O. maritimus* ticks collected in 1981 and 1982 in the nests of herring gulls on islands of South Brittany, France. No antibody to MEAV was detected in sera collected from local residents (Chastel *et al.* 1985). SREV was isolated from seabird ticks of species *O. capensis* collected from the nests of Sooty Terns in 1974 and *I. eudyptidis* collected from two dead Silver Gulls (*Larus*) in 1826 (St George *et al.* 1977). TYUV was originally isolated from *I. putus* ticks collected from rifts in rocks at a seabird colony on Tuleniy Island (Lvov *et al.* 1971). Though no human nor other mammalian animal disease were related with these S-TBFVs, all these viruses can cause paralysis in suckling mouse, and SREV can even cause death in mice (Chastel *et al.* 1985; Lvov *et al.* 1971; St George *et al.* 1977).

Kadam virus (KADV) was originally isolated from *R. pravus* ticks collected from a cow in Uganda (Henderson *et al.* 1970). Subsequently, it was isolated in ticks from the Ar-Riyadh region in Saudi Arabia, and Uganda (Al-Khalifa *et al.* 2007). KADV was initially assigned to the M-TBFV group (Labuda and Nuttall 2004), but analysis of genetic distances showed that KADV constitutes a putative third group of tick-borne viruses in addition to the M-TBFV and S-TBFV groups (Grard *et al.* 2007).

### *Orthomyxoviridae*

The family *Orthomyxoviridae* comprises viruses characterized with six to eight segments of linear, negative-sense RNA genomes (King *et al.* 2012). One most notorious representative in this family *Orthomyxoviridae* is influenza virus which attracted significant attentions from physicians and researchers (Urbaniak *et al.* 2014). In this family, two recently proposed genera, *Quarjavirus* and genus *Thogotovirus*, contain members of TBVs (Da Silva *et al.* 2005; King *et al.* 2012; Presti *et al.* 2009).

#### *Quaranjavirus*

Two species, Johnston Atoll virus (JAV) and Quaranfil virus (QRFV) are included in genus *Quaranjavirus* (Presti *et al.* 2009). JAV was originally isolated from soft ticks (*O. capensis*) collected in 1964 from a Noddy Tern (*Anous stolidus*) nest, Sand Island, Johnston Atoll in the Central Pacific (Clifford *et al.* 1968). Since then, Eastern Australia, New Zealand, and Hawaii have reported successful isolation of JAV (Austin 1978). To date, no human disease has been reported to be associated with JAV, but it is lethal to newborn mice after subcutaneous inoculation (Clifford *et al.* 1968). QRFV was first isolated in 1953 from children with mild febrile illness in Egypt. Subsequently, it was also isolated from ticks (*A. arboreus*) and birds collected in Egypt, South Africa, Afghanistan, Nigeria, Kuwait, Iraq, Yemen, and Iran (Converse and Moussa 1982; Kemp *et al.* 1975). Human serological studies showed approximately 8% of the local population had neutralizing antibodies to QRFV, suggesting the potential for infection by QRFV in humans (Taylor *et al.* 1966b). Experimental QRFV infection in laboratory mice caused a lethal respiratory disease and meningoencephalitis, indicating the pathogenic ability of ORFV infection in animals (Baskerville and Lloyd 1976). So the characteristics and pathogenesis of the novel tick-borne orthomyxoviruses require further investigations.

#### *Thogotovirus*

Thogoto virus (THOV) is the type species of genus *Thogotovirus*, and it was identified and isolated from *Boophilus* spp. and *Rhipicephalus* spp. ticks in Kenya (Africa) (Karabatsos 1978; Sang *et al.* 2006) and Sicily (Europe), from *Am. variegatum* in Nigeria, and from *Hyalomma* spp. ticks in both Nigeria and Egypt (Calisher *et al.* 1987; Karabatsos 1978). THOV infection has been reported to be pathogenic for sheep and has been associated with high level of abortion. Other livestock animals, including cattle, goats, and mongoose, can also be infected (Service 2001). Two human cases with natural infections of THOV have been reported with typical clinical manifestations including fever, encephalitis or meningoencephalitis, and one death was reported (Moore *et al.* 1975). Dhori virus (DHOV) was isolated from *Hyalomma* spp. ticks and was reported in India, eastern Russia, Egypt, and Southern Portugal (Anderson and Casals 1973; Filipe and Casals 1979; L’Vov *et al.* 2002). Five human cases with DHOV infection were identified who were febrile and had encephalitis, indicative of the potential of thogotoviruses to induce human diseases (Butenko *et al.* 1987). Jos virus (JOSV) was originally isolated from cow serum in Nigeria in 1967, and was then isolated from *Amblyomma* spp. and *Rhipicephalus* spp. ticks from Ethiopic, Guinea, Central Africa Republic, Nigeria, Ivory Coast, and Senegal (Bussetti *et al.* 2012; Karabatsos 1978). Though no diseased cases associated with JOSV have been reported for human and livestock, several studies have confirmed that JOSV could cause a fatal illness with acute hepatocellular necrosis when inoculated into newborn mice, suggestive of the potential health threat to animals (Bussetti *et al.* 2012; Fagbami and Ikede 1978).

Interestingly, thogotovirus surface glycoproteins show no similarities to any influenza viral proteins but have striking sequence homology to a baculovirus surface glycoprotein (Freedman-Faulstich and Fuller 1990; Morse *et al.* 1992). This significant difference between thogotovirus and other influenza viruses may be responsible for the ability of different host adaption in tick and avian (Freedman-Faulstich and Fuller 1990; Kimble *et al.* 2010). Significantly, the high structural and biochemical similarities of thogotovirus to influenza virus, their abundance and wide distribution in the world, and the high possibility and ability of orthomyxoviruses to undergo reassortment together make thogotoviruses deserving of more attention in the future in case of large epidemics.

### *Reoviridae*

*Reoviridae* represents the largest family of dsRNA viruses containing viruses isolated from a wide range of vertebrates, invertebrates, plants, insects, and bacteria. Virus members of *Reoviridae* have genomes composed of 9–12 segments of linear dsRNA. The virus particles have icosahedral symmetry with a diameter of approximately 60–85 nm comprising three concentric protein layers which are designated as outer capsid, subcore, and core structures from the outer layer to the inner layer, respectively (Hill *et al.* 1999; Nason *et al.* 2004). *Reoviridae* includes 75 virus species and 30 further tentative species (Brussaard *et al.* 2004). According to differences in the morphological and genetic features, *Reoviridae* is divided into two subfamilies, *Spinareovirinae* and *Sedoreovirinae*. Subfamily *Spinareovirinae* describes the genera including spiked or turreted viruses, and *Sedoreovirinae* characterizes the genera containing the non-turreted viruses with relatively smooth morphology. Fifteen genera are included in this family, six belonging to the *Sedoreovirinae* subfamily (*Cardoreovirus*, *Mimoreovirus*, *Orbivirus*, *Phytoreovirus*, *Rotavirus*, *Seadornavirus*) and nine belonging to the *Spinareovirinae* subfamily (*Aquareovirus*, *Coltivirus*, *Cypovirus*, *Dinovernavirus*, *Fijivirus*, *Idnoreovirus*, *Mycoreovirus*, *Orthoreovirus*, *Oryzavirus*). Coltiviruses belonging to the *Spinareovirinae* subfamily and orbiviruses belonging to the *Sedoreovirinae* subfamily are arboviruses including TBVs.

#### *Coltivirus*

Presently, the genus *Coltivirus* contains Colorado tick fever virus (CTFV), California hare coltivirus (CTFV-Ca, a serotype of CTFV), and Eyach virus (EYAV) (King *et al.* 2012). Coltiviruses are mainly transmitted by ticks of family Ixodidae. CTFV is the typical species of genus *Coltivirus*. It causes an endemic disease named Colorado tick fever (CTF) in humans in northwestern America. It was initially confused with a mild form of Rocky Mountain spotted fever caused by *Rickettsia rickettsii*, until in 1946 when CTFV was isolated from human serum (Florio *et al.* 1946). CTFV is transmitted mainly by the wood tick *D. andersoni*, and other ticks such as *D. occidentalis*, *D. albipictus*, *D. arumapertus*, *H. leporispalustris*, *Ot. lagophilus*, *I. sculptus*, and *I. spinipalpis* are also associated with CTFV infection (Attoui *et al.* 2005). CTFV has a wide host range including ground squirrels, chipmunks, wild mice, wood rats, wild rabbits and hares, porcupines, marmots, deer, elk, sheep, and coyotes. Patients with CTFV infection have clinical signs including an abrupt onset of fever, chills, headache, retroorbital pain, photophobia, myalgia, abdominal pain, and generalized malaise. Patients with severe symptoms may have infection of the central nervous system (CNS) or hemorrhagic fever, pericarditis, myocarditis, and orchitis, which have been mainly observed in children (Attoui *et al.* 2005). EYAV is a distinct viral species but antigenically related to CTFV (Rehse-Kupper *et al.* 1976). It was first isolated in 1976 from *I. ricinus* ticks in southwestern Germany and from *I. ricinus* and *I. ventalloi* in 1981 (Chastel *et al.* 1984). The reservoir of EYAV is thought to be the European rabbit (*Oryctolagus cunniculus*), but the natural cycle of the virus is still unclear. Serological tests have indicated that EYAV is associated with meningoencephalitis in humans (Labuda and Nuttall 2004).

#### *Orbivirus*

The genus *Orbivirus* contains 22 recognized virus species (Roy and Noad 2006). Most orbiviruses are vertebrate-infecting viruses transmitted by blood-feeding arthropod vectors (Hubalek and Rudolf 2012). Five orbivirus species are TBVs: *Chenuda virus*, *Chobar Gorge virus*, *Great Island virus*, *St Croix River virus*, and *Wad Medani virus*. These tick-borne orboviruses (TBOVs) share low level identities with and are distantly related to other orbiviruses (Belaganahalli *et al.* 2015).

The species *Chenuda virus* includes seven serotypes: Baku virus (BAKUV), Chenuda virus (CNUV), Essaouira virus (ESSV), Huacho virus (HUAV), Kala Iris virus (KIRV), Mono Lake virus (MLV), and Sixgun City virus (SCV). BAKUV was isolated in 1970 in the Soviet Union (Karabatsos 1978). CNUV was isolated in 1954 from ticks in Egypt, with serological evidence of infection in birds, camels, pigs, buffalo, dogs, donkeys and rodents (Karabatsos 1978). ESSV and KIRV were isolated from *O. maritimus* from Morocco in 1979 and 1981, respectively (Chastel *et al.* 1993). HUAV was isolated in Peru in 1967 (Clifford *et al.* 1980), while MLV and SCV were isolated in 1966 and 1969, respectively, in the United States (Calisher *et al.* 1988; Labuda and Nuttall 2004). The species *Chobar Gorge virus* is associated with bats and includes two serotypes. The prototype Chobar Gorge virus (CGV) was isolated in 1970 from *Ornithodoros* spp. ticks in Nepal. Serological evidence revealed the potential for infection by CGV to cattle, horses, sheep, buffalo, and humans (Karabatsos 1978). Presently, *Great Island virus* represents the single species containing members of 36 serotypes, which are assigned as the *GIV* group. Viral members of *GIV* group are quite widespread in Europe, North America, the Russian Far East, and a sub-antarctic island (Hubalek and Rudolf 2012; Main *et al.* 1973; Nunn *et al.* 2006). The prototype, Great Island virus (GIV), was originally isolated from the *I. uriae* ticks and the seabird host *Fratercula arctica* in Newfoundland, Canada. Neutralizing antibodies were identified in sera from other seabirds (*Uria aalge*), suggesting possible infection of GIV in seabirds (Moss *et al.* 1988; Nunn *et al.* 2006). Kemerovo virus (KEMV) was isolated from *I. persulcatus* female ticks collected in the Kemerovo region (Russia) (Libikova *et al.* 1970). Neutralizing antibodies to KEMV were found in sera from humans, livestock, wild rodents, and birds (Libikova *et al.* 1970, 1978). Another two members of the *GIV* group, Lipovnik virus (LIPV) and Tribec virus (TRBV), which are quite closely related to KEMV, were isolated from adult *I. ricinus* ticks collected in Czechoslovakia in 1963 (Libikova *et al.* 1964). In subsequent years, KEMV, LIPV, TRBV, and other viral members with similar antigenic properties were identified and isolated from ticks in different continents, which comprise the *GIV* group of up to 36 members (Dedkov *et al.* 2014). The species *Wad Medani virus* includes two serotypes: Seletar virus (SELV) and Wad Medani virus (WMV). SELV was isolated from *B. microplus* ticks collected in the Seletar district, Singapore, in 1961. Serological evidence suggested the infection in cattle, camel, pigs, buffalo and rodents (Karabatsos 1978; Taylor *et al.* 1966a). WMV was first isolated from ticks collected at Wad Medani in Sudan in 1952, and subsequently from sheep and ixodid ticks from in the Sudan, West Pakistan, India, Russia, and Jamaica. St Croix River virus (SCRV) was the first recognized endogenous virus isolated from tick cell lines (IDE2) established in 1994 from eggs of *I. scapularis* ticks (Attoui *et al.* 2001). Then, it was found in other tick cell lines, IDE8 (*I. scapularis*), and RA243 and RA257 (*R. appendiculatus*) (Alberdi *et al.* 2012). SCRV is considered as a “tick only” virus because it fails to infect any other non-tick cells including the cell lines derived from mosquitoes, amphibians, and mammals (Bell-Sakyi and Attoui 2013). Phylogenetic analysis indicates that SCRV represents a lineage ancestral to other known TBOVs (Bell-Sakyi and Attoui 2013). It strongly indicated that SCRV is unlikely to be an arbovirus.

## TBVs Unassigned to Families

Researchers are making efforts to identify, isolate, and characterize novel TBVs, and to determine whether these TBVs pose threats to public health, so that they can prepare for a rapid response to newly emerging diseases caused by novel TBVs. Taking advantage of NGS, a large number of novel virus-related sequences have been identified in different tick species; however, researchers failed to isolate these viruses (Li *et al.* 2015; Tokarz *et al.* 2014b). Phylogenetic analyses showed some of the novel TBVs are close to assigned virus families and genera, but most others are distantly related to defined families (15.8%–54.2%) and thus could not be assigned (Table 2) (Li *et al.* 2015; Qin *et al.* 2014; Tokarz *et al.* 2014b). A novel group of segmented RNA viruses was discovered taking Jingmen tick virus (JMTV) as the representative. The genome of JMTV is partly related to flaviviruses, but employs a different genome organization (Callister *et al.* 2008; Qin *et al.* 2014). The discovery of JMTV and other related segmented RNA viruses might reveal an unanticipated evolutionary link between segmented and unsegmented RNA viruses (Qin *et al.* 2014). Some other TBVs have employed different strategies for genome organization like circular genomes and segmented circular genomes, which have been rarely described before. These viruses locate between the segmented and unsegmented linear RNA viruses according to their phylogenic relationships (Li *et al.* 2015). All these data have shown the great diversity of TBVs and indicated there might be much more diversity than we previously thought.

## Conclusion

Since the discovery of first tick-borne pathogenic virus over 100 years ago, diversified TBVs with global distribution have been discovered and isolated belonging to at least 2 orders, 9 families, and 12 genera. In recent years, the rapid development of NGS has boosted the discovery of novel TBVs. An unexpected role of TBVs in the evolutions of RNA viruses has been revealed, which also suggests the significant role of ticks in circulating and transmitting TBVs. The known TBVs may just represent a tip of the iceberg, the most and rest part of which remains to be explored.

Our current knowledge about the association of TBVs and tick species are still limited. Hopefully, ongoing researches from worldwide scientists on the immunomodulation mechanism of tick salivary, interactions between tick viruses and the transmitted-vector tick species, and anti-tick vaccines will have great significance in controlling of tick-borne viruses and protecting humans and livestock from pathogenic TBVs.

## Abbreviations of generic name of tick

*A*: *Argas*
*Am*: *Amblyomma*
*B*: *Boophilus*
*D*: *Dermacentor*
*Hy*: *Hyalomma*
*H*: *Haemaphysalis*
*I*: *Ixodes*
*O*: *Ornithodoros*
*Ot*: *Otobius*
*R*: *Rhipicephalus*
